# Supplementary material for: The interactome of intact mitochondria by cross-linking mass spectrometry provides evidence for coexisting respiratory supercomplexes
Source: Mol Cell Proteomics. 2017 Dec 8;17(2):216–32. doi: 10.1074/mcp.RA117.000470 (PMC5795388; doi:10.1074/mcp.RA117.000470)
Supplement: Supplemental Data [file supp_RA117.000470_133922_0_supp_23978_fzffwf.zip › spectra_annotation/mito_spectra_annotation/359-1-67-1-7-1.pdf]

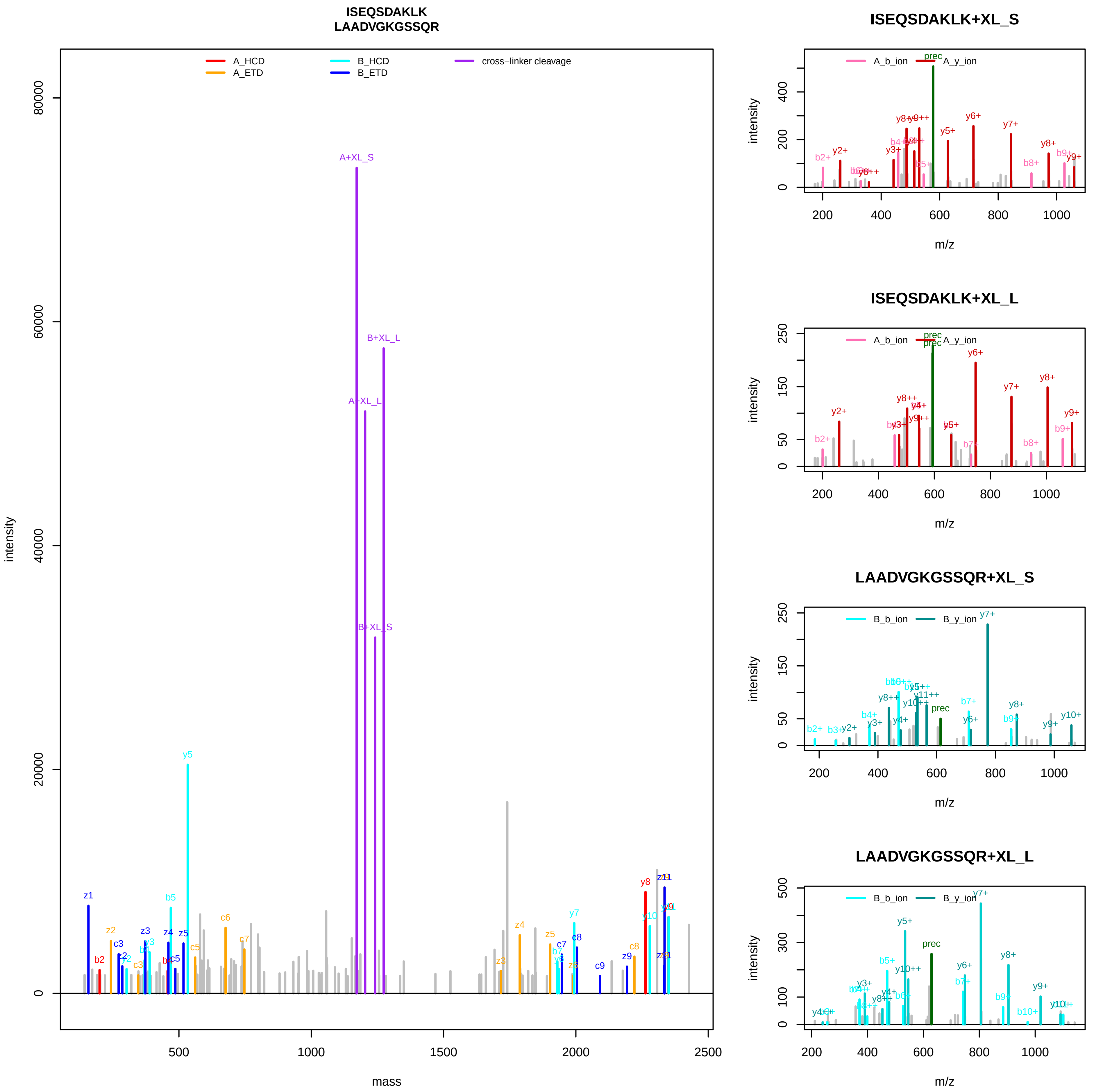

ISEQSDAKLK
LAADVGKGSSQR
A_HCD
A_ETD
B_HCD
B_ETD
cross−linker cleavage
A+XL_S
B+XL_L
A+XL_L
B+XL_S
intensity
mass
0
20000
40000
60000
80000
500
1000
1500
2000
2500
z1
b2
z2
c3
y2
c3
z3
y3
b4
z4
z5
c5
b5
y5
c5
c6
c7
z3
z4
z5
c7
c8
y7
c9
z9
c8
y8
y10
z11
y9
ISEQSDAKLK+XL_S
ISEQSDAKLK+XL_L
LAADVGKGSSQR+XL_S
LAADVGKGSSQR+XL_L
A_b_ion
A_y_ion
B_b_ion
B_y_ion
prec
m/z
intensity
b2+
y2+
y3+
y5+
y6+
y7+
y8+
y9+
b8+
b9+
y7+
y8+
y9+
y10+
b7+
b9+
